# Perception of Illness and Fear of Inhaled Corticosteroid Use among Parents of Children with Asthma

**DOI:** 10.3390/children10101597

**Published:** 2023-09-25

**Authors:** Jasna Petric Duvnjak, Emilija Lozo Vukovac, Anita Ursic, Antonela Matana, Ivana Medvedec Mikic

**Affiliations:** 1 Pediatric Clinic “Pediatri”, 21000 Split, Croatia; jpetric@mefst.hr (J.P.D.); anita.ursic@mefst.hr (A.U.); 2 University Department of Health Studies, University of Split, 21000 Split, Croatia; antmatana@ozs.unist.hr; 3 School of Medicine, University of Split, 21000 Split, Croatia; elozovuk@mefst.hr; 4 Department of Pulmonary Diseases, University Hospital of Split, 21000 Split, Croatia; 5 Department of Maxillofacial Surgery, University Hospital of Split, 21000 Split, Croatia

**Keywords:** asthma, anti-asthmatic agents, inhalation drug administration, pediatrics, fear of drug use

## Abstract

The most prevalent children’s chronic disease worldwide is asthma which has notable negative impacts on patients’ and parent’s quality of life. Daily inhaled corticosteroids (ICS) therapy is a preferred controller choice. This study was conducted on 148 parents of asthmatic children to establish parents’ perception of illness and fear of inhaled corticosteroids using B-IPQ and TOPICOP questionnaires. Children were in the majority male (66.9%), older than five years (58.8%), with comorbidities, and family history of atopy. Parents were female, with a mean age of 38, employed, and with a history of some form of corticosteroid use. Most parents were not afraid of ICS usage (71.6%). Unemployed parents and parents who had no medical education had a statistically significantly higher fear of using ICS (*p* = 0.002, *p* = 0.03). A child’s illness affects the parents’ lives and parents who are afraid of using ICS react more emotionally to the child’s illness. Better understanding and less concerned about child’s disease are parents of children with controlled asthma. The parents’ perspective of children’s asthma will affect the duration and dose of ICS treatment they will give to their children and directly influence the level of asthma control.

## 1. Introduction

The most prevalent chronic disease among children worldwide is asthma [1]. According to the World Health Organization (WHO), 262 million people globally suffer from asthma [2]. In 2019, according to Global Burden of Disease (GBD) data, 6.16% of the population suffered from asthma [3]. In Croatia, the prevalence of asthma was 4.61% for all ages and 4.66% for children younger than 14 years [3]. Asthma is usually described as a heterogeneous illness with many different clinical phenotypes such are allergic or non-allergic asthma, among others [4,5]. Recurrent respiratory symptoms like shortness of breath, cough, or wheezing, chest tightness, together with different degrees of expiratory airway flow limitation are defined as asthma. Patients suffer from exacerbation that sometimes can be resolved without medication, but on the other hand, can be fatal. 

Regular, daily inhaled corticosteroids (ICS) are a preferred controller choice [6], and even with low daily doses, it would impact asthma hospitalizations and deaths [7]. The minimum effective dose of ICS is recommended to avoid possible corticosteroid side effects [8]. Yet, with an excellent safety profile and well-documented suppression of airway inflammation [9], ICS also have local and systemic side effects [10]. 

Side effects of ICS are dose-reliant and more often occur in high-dose corticosteroid asthma users [11]. Some local side effects like oral candidiasis, dental caries, and dysphonia can be avoided with better inhaler technique, valve chamber use, and rinsing a mouth and face with water after corticosteroid inhalation [10]. One of the major parent’s concerns is the impact of inhalational corticosteroid use on their children’s linear growth; suppressed growth velocity or reduced final height, an effect that may be a delivery device- or drug molecule-dependent [12,13]. The fact that uncontrolled asthma and atopy have a negative influence on linear growth too must be presented during pediatric asthma visits [14]. High-dose ICS use may cause adrenal suppression and lead to adrenal insufficiency under stress or therapy withdrawal [15]. Given the possible side effects, the concern of parents when using ICS for asthma therapy and control is understandable. 

Even though reasons are still not completely understood, the ICS adherence rate in children with asthma is only 50% [16]. Poor ICS adherence is a potentially modified risk factor for exacerbation, even in patients with few asthma symptoms [17], so it is important to enlighten parents about that fact.

Perceptions of chronic diseases are a driver of patient behavior and may have an influence on treatment outcomes [18]. Parental decision to give a child prescribed ICS is based on their perception of asthma and can be modified through partnership with a clinician [19]. According to recent literature, fear of using ICS is documented in up to 67.3% of families with children suffering from asthma [13]. To deal with this important adherence obstacle, clinicians must identify, in every individual family parents’, the main beliefs and fears about prescribed ICS. 

Parents of children with asthma have a primary responsibility to monitor for symptoms and properly administer controller therapy [20]. Parents’ goals, preferences, and concerns about disease and medication must be communicated as a means to promote their active role [21]. Good communication strategies between experts and parents will help to achieve the much-needed partnership necessary for successful treatment [22] and improved outcomes of child asthma [23,24,25]. Training parents to ask information and seek clarification in any doubt about treatment will result in better medication adherence [26]. 

This study aimed to gain insight into parents’ perception of asthma, their fears, and beliefs about using ICS in 2–10-year-old children using two questionnaires, TOPICOP and B-IPQ. The hypothesis of this study was that the parents of children with well-controlled asthma will have less fear and concerns about ICS and have a positive influence on ICS treatment adherence.

To our knowledge, this is the first cross-sectional research using these two questionnaires for this particular purpose and population. 

## 2. Materials and Methods

### 2.1. Study Design

This cross-sectional questionnaire-based study was conducted from April 2023 to July 2023 in Split, Croatia. The parents and children during regular, prescheduled, asthma-related visits to pediatric pulmonologists were enrolled in this study. They filled out the online form of the questionnaires while waiting in the waiting room on the tablet. The questionnaire had three parts: demographic data, the perception of the child’s illness (Brief Illness Perception Questionnaire (B-IPQ), and questions about beliefs and worries about ICS use (TOPICOP questionnaire). From the medical records of the children, we had data on age, gender, age of disease onset, allergic sensitization, comorbidities, IgE level, and family history. The level of asthma symptom control for 4-week prior visit was assessed by a pediatric pulmonologist based on GINA guidelines [8]. Symptoms of asthma during the day or night, the child’s level of activity, and the need for reliever therapy of short-acting beta agonist (SABA) were scored. In this study, we included parents or legal guardians of children younger than 18 years of age, previously diagnosed with asthma according to the GINA guidelines by the pulmonologist, and at least 3 months prescribed daily ICS. Patients with acute asthma exacerbation or other acute illnesses and patients treated with ICS for other causes than asthma did not participate in the study. All subjects signed informed consent before they participated in the study. The study was carried out following the Declaration of Helsinki, and the Ethics Committee of the School of Medicine, University of Split (003-08/23-03/0015, 20 April 2023) approved the protocol.

### 2.2. Demographic Information

The demographic data included parents’ age, gender, qualifications, employment status, general health status, number of children, history of smoking, history of asthma or any type of allergy, and history of corticosteroid usage. 

### 2.3. The Brief Illness Perception Questionnaire (B-IPQ)

In this study, B-IPQ [27] was translated into Croatian language, adapted for parents of children with asthma, and validated as having acceptable psychometric properties. Cronbach’s Alpha was 0.685, indicating acceptable reliability. This eight-item scale was made to quickly assess the emotional and cognitive representations of illness on a ten-point Likert scale. The main preferences offered by the Brief IPQ to researchers were shortness and velocity of completion for patients and the lightness of interpretation of scores. Five items assessed cognitive disease representations: consequences (Item 1), timeline (Item 2), personal control (Item 3), treatment control (Item 4), and identity (Item 5). Two items estimated emotional representations: concern (Item 6) and emotions (Item 8). One item estimated disease comprehensibility (Item 7). The eight questions were (I) how much does your child’s illness affect your life, (II) how long do you think the illness will continue, (III) how much control do you feel they have over your child’s illness, (IV) how much do you think treatment can help your child’s illness, (V) how much do you experience symptoms from your child’s illness, (VI) how concerned are you about your child’s illness, (VII) how well do you feel that you understand your child’s illness, (IX) how much does your child’s illness affect you emotionally (makes you mad, worried, upset, or sad). Items were assigned from 0 to 10. The higher sum reflected poorer perception of children’s asthma. 

### 2.4. TOPICOP Questionnaire

For the cause of this study, the TOPICOP questionnaire [28] was translated into Croatian, adapted for use by parents of children with asthma, and validated. The internal consistency between all 10 items was α = 0.743, indicating acceptable reliability. The evaluation of inhaled corticosteroids (ICS) fear was quite an important step in the management of patients with asthma. The questionnaire TOPICOP (topical corticophobia) was established to assess the patients and their parent’s fears and beliefs about the use of topical CS. This questionnaire can help researchers and clinicians to better understand what has an influence on therapeutic adherence. The first self-administered scale, TOPICOP, was developed to evaluate AD patients’ or their parent’s fear of corticosteroids (CS). It contained statements; five related to fears and beliefs and exhibited great psychometric properties. The statements were simple and easily understood.

Three response choices were offered, from I do not agree to I totally agree, with points attributed to each one (0, 1, or 2), with higher values corresponding to a more severe fear of CS. The maximum number obtained based on the answers to the questionnaire was 20 (10 questions of 2 points each). Instead of CS in the questionnaire, we used ICS (inhalation corticosteroids). On the basis of the questionnaire, parents were divided into two groups: “afraid of ICS” and “not afraid of ICS”.

### 2.5. Statistical Analyses

For normality checking, the Kolmogorov–Smirnov test was used. Continuous variables are presented as the median (interquartile range, IQR) due to the non-normal distribution of the data. Categorical variables are expressed with frequencies (percentages). Differences in categorical variables were analyzed by using Fisher’s exact and Chi-square test. Mann–Whitney and Kruskal–Wallis tests were used for not normally distributed continuous variables [29]. To analyze correlations between not normally distributed variables, the Spearman rank correlation test was used. We also performed a multivariate logistic regression analysis to examine factors that were associated with fear of using ICS. Odds ratios (OR) and 95% confidence intervals (CI) were calculated. Statistically significance was considered with two-sided *p*-values less than 0.05. Statistical analysis was conducted using Statistical Package Software for Social Science, version 28 (SPSS Inc., Chicago, IL, USA).

## 3. Results

### 3.1. Children’s Descriptive Features

This study involved a group of 148 children with asthma; 99 (66.9%) of them were male. The median age was 5.65 (IQR: 5.52, 2 to 10 years old). They were divided into two groups: younger than five years, 61 (41.2%), and older than five years, 87 (58.8%). According to medical records, more than half of respondents had allergic sensitization to aeroallergens and one-quarter to food allergens. Almost all children had a family history of atopy (atopic dermatitis, allergic rhinitis, food allergy). More than half of the children did not have a pet (bird, cat, dog, or hamster). In addition to asthma, three-quarters of children had comorbidities (atopic dermatitis, food allergy, drug allergy, allergic rhinitis). Almost 40% of children had to visit the emergency service due to asthma, while one-fifth of them were hospitalized; 3 were hospitalized in the last 4 weeks. Only 38.5% of all children experienced asthma symptom control 4 weeks before the visit. Among all the respondents, more than half played sports. Alternative treatment (donkey milk, black seed oil, probiotics, vitamin D) was used by more than 70% of respondents (Table 1). Asthma symptom control was assessed according to the GINA assessment and included questions about the last four weeks’ daytime and nighttime symptoms, activity limitation, and reliever (SABA) medication use. A well-controlled level of asthma symptom control means that in the last four weeks, the child did not experience any daytime symptoms longer than a few minutes, any activity limitation (running, playing), any night waking or coughing, or using SABA more than once a week for children 5 years and younger or more than twice a week for children 6 years old and older. The use of reliever before exercise was excluded from the assessment. 

### 3.2. Parents Descriptive Characteristics

The average age of correspondent parents or legal guardians was 38 (ranging from 26 to 67), and all of them were female. Most parents finished high school, more than a half were employed, had two children, and rated their health as 4-very good (scale from 1—bad to 5—excellent). Less than 15% of parents have any kind of medical education and almost a third of them declared themselves as smokers. A small number of parents reported a history of asthma and atopic dermatitis (AD), but 30.4% have allergic rhinitis (AR). Most parents did not ever use inhaled or oral corticosteroids, but almost half of them had used topical corticosteroids (Table 2).

### 3.3. TOPICOP Questionnaire Result

The results obtained from the TOPICOP showed that almost half of the parents did not believe that ICS makes their children fat (50%). Most of them did not believe that ICS usage can lead to infections (66.9%), partially agreed with the statement ICS passes into the bloodstream (46.6%), did not agree that ICS can damage children’s lungs (58.1%), and partially agreed that ICS will affect their child’s health (56.8%). As for fears, the majority of parents want to be informed about the medicines their child is taking (80.4%), and most of them partially agreed with the statement that they want their child to stop taking ICS therapy as soon as possible (48.6%), and most of them are afraid of using more medicines (higher ICS dose) (42.6%). A significant number of parents (44.6%) did not agree with the statement that they are afraid of using ICS though they are not familiar with the side effects of this drug. More than half of the parents (54.1%) wait as long as possible before using ICS (Table 3).

### 3.4. Parent Distribution Based on Results of the TOPICOP Questionnaire

Parents with TOPICOP scores > 10 are afraid of ICS usage and there were 42 (28.4%) of them. The rest of the 106 (71.6%) parents had TOPICOP scores < 10 and they had no fear of ICS usage.

We did not detect statistically significant associations between parent’s fear of using ICS and various variables in children with asthma, as shown in Table 4. 

However, even though it was not statistically significant, it can be noted that parents of male children, older than 5 years, who are sensitized on aeroallergens, have a positive family history of atopy, have comorbidities, children who have pets and who have been hospitalized because of asthma, and children who are receiving alternative therapy have a greater fear of using IC.

Comparing the demographic data on the our participants who filled out the questionnaires using univariate statistical analysis, statistically significant differences were obtained between the group of parents who were afraid of using ICS and those who were not. Unemployed parents and parents who had no medical education had a statistically significantly higher fear of using ICS than the other group of parents. As shown in Table 5, other variables did not show statistical significance. The fear of using ICS was greater among parents who have never used ICS or topical corticosteroids. The situation was reversed for parents who did not use oral corticosteroids. Furthermore, we performed a multivariate logistic regression analysis for fear of using ICS with 13 predictors listed in Table 5 (*p* = 0.034, Nagelkerke R2 = 0.223). Statistical significance was observed for the employment status of parents, i.e., unemployed parents had a higher fear of using ICS than employed parents. Other variables did not show statistical significance.

### 3.5. The Results of the B-IPQ Questionnaire 

Looking at the results of the B-IPQ questionnaire (Table 6), we can see that the child’s illness affects the parents’ lives, the parents think that the illness will last for some time, they think that they have very good control over the child’s illness, and that the therapy helps their child very well. They stated that the symptoms are mostly moderately serious, and they are quite worried about the child’s illness. They understand their child’s illness very well, and their child’s illness affects them emotionally. 

Comparing the results of the B-IPQ questionnaire between the two groups; “fear of using ICS” and “no fear of ICS”, a statistically significant difference was found in two questions—“To what extent do you experience the symptoms of your child’s illness?” and “How worried are you about your child’s illness?”. Parents in the group of fear the use of ICS perceived the symptoms of their child’s illness more seriously (*p* = 0.026) and were more concerned about treatment (*p* = 0.040). Although it was not statistically significant, a difference was noticed in the impact of the child’s disease on the parent’s emotional state. Parents who were afraid of using ICS react more emotionally to their child’s illness (*p* = 0.063). The other results were not statistically significant.

### 3.6. Correlation between TOPICOP and B-IPQ Results

Studying the correlation between the components of the TOPICOP and the B-IPQ questionnaires, statistically significant positive correlations were found between the duration of the disease (B-IPQ 2) and the opinion that the use of ICS will affect the child’s future health (r = 0.198; *p* = 0.016) and between the understanding of the child’s illness (B-IPQ7) and the desire for more information about ICS (r = 0.177; *p* = 0.032). Negative correlations were recorded comparing the control of the child’s illness (B-IPQ3) and the desire to discontinue ICS therapy (r = −0.227; *p* = 0.006) and the use of higher ICS dose (r = −0.257; *p* = 0.002). Between the opinion of how much treatment can help your child (B-IPQ4) and the desire to stop ICS therapy as soon as possible (r = −0.332; *p* < 0.001), as well as between the same B-IPQ4 component and the fear of taking too many drugs in general (r = −0.248; *p* = 0.002) a negative correlation was found. The degree of understanding of the child’s illness (B-IPQ7) and the desire to stop ICS therapy as soon as possible (r = −0.023; *p* = 0.013) and the fear of taking a higher ICS dose (r = −0.193; *p* = 0.019) were also negatively correlated.

Comparing the results of the B-IPQ questionnaire and the degree of asthma symptom control, statistically significant positive correlations were seen between the questions “To what extent does your child’s illness affect your life?” (*p* = 0.004) and “How well do you understand your child’s illness?” (*p* = 0.035). Borderline statistical significance was found for the question “How worried are you about your child’s illness?” (*p* = 0.054).

## 4. Discussion

As previous studies about the natural history of asthma suggested, asthma symptoms start at an early age tend to continue in true adulthood [30]. According to available literature, 80% of asthma starts up to the fifth year of the child’s life [31], with the predominance of male gender until the age of 10 [32]. Our respondents were also male children slightly older than five years since it is the most frequent group of patients with asthma in our pediatric pulmonologist practice. 

Sensitization to common aeroallergens of children with asthma involved in this study was 52%, which was assessed with an allergen-specific immunoglobulin E (IgE) level or skin prick test. Similar numbers were published by Arbes et al. in their research where 56.3% of asthma patients between 6 and 59 years were attributed to atopy [33]. The literature indicates that early-age allergic sensitization is a major predictor for persistent asthma development [34]. 

Almost all children of our participants had a positive family history of atopic dermatitis, allergic rhinitis, or food allergies. Only 14.9% of parents had a history of asthma, but among all members of the family, a history of asthma was observed in 69.6% of cases. The results emphasize the need to take a wider family history because the possibility of asthma development grows with the number of family members with asthma [35]. Almost three-quarters of the children of our respondents had comorbidities (allergic rhinitis, atopic dermatitis, food or drug allergy), that can be easily explained by “atopic marsh” development [36]. Physical activity is one of the non-pharmacological treatments of asthma because of its impact on general health [9]. Around half of the observed children with asthma in this study were involved in some kind of organized sports activity which is encouraging proof of a healthy lifestyle. There was a small number families with household pets, mostly dogs and cats, which is desirable and recommended for these allergen-sensitized children [37]. A recent meta-analysis by Ji et al. showed a significant occurrence of childhood asthma in dog and cat owners [38]. 

As previously reported, mothers have a critical role in their children’s quality of life and development of anxiety [34], one of the “treatable traits” [39], and risk factors for asthma exacerbation [8]. In our study, the questionnaires were, in 99 percent of cases, answered by mothers who were mostly high school educated and employed.

To examine the fear of parents about ICS usage and divide them into two groups; “afraid” and “not afraid of ICS treatment”, we used the TOPICOP questionnaire similar to Choi et al. [40]. While the question of adherence obstacles and thoughts to corticosteroid therapy is usually discussed during asthma child visits, the roots of these worries are rarely explored. It can be based on personal experience, non-medical information from family, friends, social media [41], or other parents of asthmatic children, and sometimes confusing information from medical professionals [42].

The TOPICOP scale was constructed to make it easier for clinicians to identify beliefs and worries for that specific person and specific health problems, and direct conversation to resolve it and improve their treatment adherence [28]. Our respondents were mostly afraid of using higher ICS doses, and totally or partially agreed to stop practicing ICS as soon as possible in around 70% of cases, higher than one-third of the parents previously observed by Yoos et al. [43]. Based on the TOPICOP questionnaire, 28.4% of our respondents were afraid of using ICS and two-thirds were concerned about steroid’s side effects. An Israelian study reported fears and concerns about ICS in 30.4% of mothers of children with asthma [44]. A much higher percentage of corticophobia group was reported in adult asthma patients (52.6%) [45]. The same authors reported that 67.3% of respondents believed that ICS may damage the lungs. Only 5% parents of our respondents shared the same fear. Around half of adult asthma patients believe that ICS will make them fat [45], while only around 10% of parents in our study had the same opinion.

Comparing the data from the children’s medical records and two groups of parents (those who are afraid of using ICS and those who are not), we see those parents of male children older than five years, with comorbidities, and previous hospitalization due to asthma have a greater fear of using ICS. This same group of parents is more inclined to use complementary and alternative therapy with donkey milk, black seed oil, vitamin D, etc., which are perceived as safe alternatives to corticosteroid treatment. The same was noticed in the work of Lee et al. on parents of children with AD [46]. In our study, unemployed parents without medical education were more afraid of using ICS. These results are in accordance with those reported by Bos et al., who stated that medically educated parents of AD children have significantly less fear of corticosteroid use [47]. Fear of ICS usage is even stronger considering that parents are often warned about the side effects of corticosteroid use by social media, friends, family members, and medical professionals (pharmacists and physicians) [42]. The impact of employment status on fear of using ICS is still not established, yet evidence from the literature suggests an important role of parents, especially mothers’ employment status, on general child health [48]. Many children in this study were treated for asthma at the emergency room, and parents of those children showed an increased fear of using ICS. Parents who had not personally used corticosteroids had a greater fear of using topical or inhaled corticosteroids than oral ones. Perhaps the fear of corticosteroids is device-dependent and related to the route of administration [13,49]. 

To examine how well parents know their child’s illness, to what extent it affects their life, and how well they think they could control it, the B-IPQ questionnaire was used. The results of this study showed that the child’s illness affects the parents’ life, also emotionally, causing concern, anger, and helplessness; this is more than in adult asthmatic patients from the original Broadbent et al. study [27]. Parents in this study were aware of the chronic character of the disease and believed that the illness will last for some time. They felt that they understood their child’s disease well, they were controlling it well, and believed that ICS therapy was helping their children. Parents who have a fear of using ICS perceive their child’s illness more seriously, are more worried about therapy, and react more emotionally to their child’s illness. Similar results can be found in the study of Conn et al. [50], who reported that parents who rated their children’s asthma attacks as more severe have more concerns about therapy. Parents who feel that they have less control over their child’s illness and treatment want to terminate using ICS as soon as possible. They are reluctant to use a higher dose of ICS significantly more than other interrogated parents. Based on that, we must make an impact on parents’ confidence in treating child asthma, because less confident parents tend to reduce ICS dose and are more concerned about addiction and toxicity of ICS [44]. The obtained results present parents believing that longer asthma duration will result in more side effects of ICS. That can be explained by the burden of “chronicity” and is similar to reports that parents better perceive short-term OCS therapy [49]. 

Based on the mentioned beliefs and parents’ fear of using ICS, it is highly recommended to clearly explain the therapy plan to the parents, set short- and long-term goals, and duration of the therapy during asthma-related pediatric visits [8]. This will result in better adherence and better child asthma control. 

Comparing the results of the child’s illness control and the parent’s perception of the disease, we can conclude that asthma affects the lives of parents whose children have uncontrolled asthma the most. The same conclusion was presented in the research of Banjari et al. [50]. Parents of children with controlled asthma better understand and they are less concerned about their child’s disease. 

## 5. Conclusions

Fully aware of the limitations of this study (one center research and pediatric pulmonologist visits), we can conclude that parent’s perspective of children’s asthma will affect the duration and dose of ICS treatment that they will give to their children and directly influence ICS treatment adherence and level of asthma control. Applying simple tools like these questionnaires will help clinicians detect the main points of perception of child asthma, fears, and beliefs about ICS treatment and break this circle.

## Figures and Tables

**Table 1:** Descriptive characteristics of the pediatric patients with asthma (n = 148).

	Descriptive Statistics (N/%)
Age	
<5	61 (41.2%)
≥5	87 (58.8%)
Gender	
Male	99 (66.9%)
Female	49 (33.1%)
Allergic sensitization to aeroallergens	
Yes	77 (52%)
No	59 (39.9%)
Allergic sensitization to food allergens	
Yes	25 (16.9%)
No	101 (68.2%)
Pets (dog, cat, bird, hamster)	
Yes	49 (33.1%)
No	99 (66.9%)
Family history of atopy	
Yes	136 (91.9%)
No	12 (8.1%)
Comorbidities	
Yes	112 (75.7%)
No	36 (24.3%)
Hospitalization due to asthma ever	
Yes	30 (20.3%)
No	118 (79.7%)
Sports	
Yes	54 (54.1%)
No	80 (36.5%)
Hospitalization last 4 weeks	
Yes	3 (2%)
No	144 (97.3%)
ER visits for asthma ever	
Yes	58 (39.2%)
No	90 (60.8%)
Alternative treatments	
Yes	112 (75.7%)
No	36 (24.3%)
Asthma symptom control	
Well-controlled	57 (38.5%)
Partly controlled	47 (31.8%)
Uncontrolled	44 (29.7%)

N—number, %-percentage, ICS—inhaled corticosteroids, ER—emergency room.

**Table 2:** Descriptive characteristics of the parents of pediatric patients with asthma (n = 148).

	All Parents (n = 148)
Age	38.03 ± 6.22
Education level	
Primary school	0
High school	64 (43.2%)
Bachelor’s degree	26 (17.6%)
Master’s degree + PhD	58 (39.2%)
Employment status	
Unemployed	32 (21.6%)
Employed	115 (77.7%)
Pensioner	1 (0.7%)
Health condition	4 (IQR:1)
Number of children	2 (IQR:1)
Medical Education	
Yes	22 (14.9%)
No	126 (85.1%)
Smoker	
Yes	41 (27.7%)
No	107 (72.3%)
History of asthma?	
Yes	22 (14.9%)
No	126 (85.1%)
History of AD?	
Yes	23 (15.5%)
No	125 (84.5%)
History of AR?	
Yes	45 (30.4%)
No	103 (69.6%)
ICS treatment	
Yes	40 (27%)
Never	108 (73%)
TCS treatment	
Yes	63 (42.6%)
Never	85 (57.4%)
OCS treatment	
Yes	37 (25%)
Never	111 (75%)

AD—atopic dermatitis, AR—allergic rhinitis, ICS—inhaled corticosteroids, TCS—topical corticosteroids, OCS—oral corticosteroids.

**Table 3:** Results of TOPICOP questionnaire.

TOPICOP Item	I Do Not Agree	Partially Agree	I Totally Agree
Beliefs			
ICS makes you fat	74 (50%)	59 (39.9%)	15 (10.1%)
ICS can lead to infections	99 (66.9%)	45 (30.4%)	4 (2.7%)
ICS passes into the bloodstream	52 (35.1%)	69 (46.6%)	27 (18.2%)
ICS damage your lung.	86 (58.1%)	55 (37.2%)	7 (4.7%)
ICS will affect my child’s health in the future	52 (35.1%)	84 (56.8%)	12 (8.1%)
Fears			
I need to be informed about medicines	2 (1.4%)	27 (18.2%)	119 (80.4%)
I want my child to stop taking the ICS as soon as possible	44 (29.7%)	72 (48.6%)	32 (21.6%)
I am afraid to use more drugs	26 (17.6%)	59 (39.9%)	63 (42.6%)
I don’t know of any side effects, but I am still afraid of ICS	66 (44.6%)	48 (32.4%)	34 (23%)
I wait as long as possible before using an inhaled corticosteroid to treat my child	80 (54.1%)	43 (29.1%)	25 (16.9%)

**Table 4:** Associations between parent’s fear of using ICS and observed variables in children with asthma.

	Parents Afraid of ICS	Parents Not Afraid of ICS	*p*-Value
Age			0.392
<5	15 (35.7%)	46 (43.4%)	
≥5	27 (64.3%)	60 (56.6%)	
Gender			0.443
Male	26 (61.9%)	73 (68.9%)	
Female	16 (38.1%)	33 (31.1%)	
Allergic sensitization to aeroallergens			0.574
Yes	25 (61%)	52 (54.7%)	
No	16 (39%)	43 (45.3%)	
Allergic sensitization to food allergens			1
Yes	7 (19.4%)	18 (20%)	
No	29 (80.6%)	72 (80%)	
Pets (dog, cat, bird, hamster)			0.460
Yes	30 (71.4%)	69 (65.1%)	
No	12 (28.6%)	37 (34.9%)	
Family history of atopy			0.108
Yes	41 (97.6%)	95 (89.6%)	
No	1 (2.4%)	11 (10.4%)	
Comorbidities			0.346
Yes	34 (81%)	78 (73.6%)	
No	8 (19%)	28 (26.4%)	
Hospitalization due to asthma			0.259
Yes	11 (26.2%)	19 (17.9%)	
No	31 (73.8%)	87 (82.1%)	
Sports			0.788
Yes	16 (42.1%)	38 (39.6%)	
No	22 (57.9%)	58 (60.4%)	
Hospitalization last 4 weeks			0.130
Yes	2 (4.9%)	1 (0.9%)	
No	39 (95.1%)	105 (99.1%)	
ER visits for asthma ever			0.209
Yes	14 (33.3%)	44 (41.5%)	
No	28 (66.7%)	62 (58.5%)	
Alternative treatments			0.605
Yes	33 (78.6%)	79 (74.5%)	
No	9 (21.4%)	27 (25.5%)	

ICS—inhaled corticosteroids, ER—emergency room.

**Table 5:** Associations between the demographic data of parents of children with asthma and fear of using ICS.

	All Parents (n = 148)				
			Univariate Analysis	Multivariate Logistic Regression Analysis	
	Afraid of ICS (n = 42)	Not Afraid of ICS (n = 106)	*p*-Value	OR (95% CI)	*p*-Value
Age	38.71± 5.58	37.75± 6.48	0.37 *	1.03 (0.96, 1.11)	0.407
Education level			0.412 **		
High school	20 (47.6%)	44 (41.5%)		1.402 (0.54, 3.63)	0.486
Bachelor degree	9 (21.4%)	17 (16%)		1.96 (0.61, 6.28)	0.257
Master’s degree + PhD	13 (31%)	45 (42.5%)		-	-
Employment status			0.002 **		
Unemployed + Pensioner	17 (40.5%)	16(15.1%)		3.48 (1.34, 8.99)	0.01
Employed	25 (59.5%)	90 (84.9%)		-	-
Health condition	4 (IQR:1)	4 (IQR:1)	1 ****	1.30 (0.76, 2.23)	0.341
Number of children	2 (IQR:1)	2 (IQR:1)	0.37 ****	0.560 (0.31, 1.01)	0.055
Medical Education			0.03 **		
Yes	2 (4.8%)	20 (18.9%)		-	-
No	40 (95.2%)	86 (81.1%)		3.59 (0.73, 17.73)	0.117
Smoker			0.578 ***		
Yes	13 (31%)	28 (26.4%)		-	-
No	29 (69%)	78 (73.6%)		0.83 (0.34, 2.06)	0.693
History of asthma?			0.25 ***		
Yes	4 (9.5%)	18 (17%)		-	-
No	38 (90.5%)	88 (83%)		0.876 (0.19, 3.96)	0.863
History of AD?			0.791 ***		
Yes	6 (14.3%)	17 (16%)		-	-
No	36 (85.7%)	89 (84%)		0.905 (0.25, 3.27)	0.879
History of AR?			0.059 ***		
Yes	8 (19%)	37 (34.9%)		-	-
Never	34 (81%)	69 (65.1%)		2.06 (0.72, 5.90)	0.176
ICS treatment			0.169 ***		
Yes	8 (19%)	32 (330.2%)		-	-
Never	34 (81%)	74 (69.8%)		1.43 (0.42, 4.86)	0.566
TCS treatment			0.289 ***		
Yes	15 (35.9%)	48 (45.3%)		-	-
Never	27 (64.3%)	58 (54.7%)		1.35 (0.49, 3.68)	0.562
OCS treatment			0.528 ***		
Yes	12 (28.6%)	25 (23.6%)		-	-
Never	30 (71.4%)	81 (76.4%)		0.53 (0.18, 1.53)	0.239

AD—atopic dermatitis, AR—allergic rhinitis, ICS—inhaled corticosteroids, TCS—topical corticosteroids, OCS—oral corticosteroids; OR—odds ratio, 95% CI—95% confidence interval; *—*t*-test, **—Fischer’s test, ***—Chi-square test, ****—Mann–Whitney test.

**Table 6:** Results of the B-IPQ questionnaire.

Item	Median (IQR)
Impact of disease on life (IPQ1)	6 (5)
Duration of illness (IPQ2)	5 (3)
Control of illness (IPQ3)	8 (4)
Treatment of illness (IPQ4)	10 (2)
Symptoms of illness (IPQ5)	6 (3)
Worry about treatment (IPQ6)	8 (3)
Understanding illness (IPQ7)	9 (3)
Impact of illness on emotional state (IPQ8)	8 (5)

IQR—interquartile range.

## Data Availability

The data presented in this study are available on request from the corresponding author. The data are not publicly available due to protection of the minor children and their parents.
